# The NWRD Dataset: An Open-Source Annotated Segmentation Dataset of Diseased Wheat Crop

**DOI:** 10.3390/s23156942

**Published:** 2023-08-04

**Authors:** Hirra Anwar, Saad Ullah Khan, Muhammad Mohsin Ghaffar, Muhammad Fayyaz, Muhammad Jawad Khan, Christian Weis, Norbert Wehn, Faisal Shafait

**Affiliations:** 1 School of Mechanical and Manufacturing Engineering, National University of Sciences & Technology, Islamabad 44000, Pakistan; jawad.khan@smme.nust.edu.pk; 2 School of Electrical Engineering and Computer Science, National University of Sciences & Technology, Islamabad 44000, Pakistan; skhan.bee20seecs@seecs.edu.pk; 3 Microelectronic Systems Design Research Group, University of Kaiserslautern-Landau, 67663 Kaiserslautern, Germany; ghaffar@eit.uni-kl.de (M.M.G.); weis@eit.uni-kl.de (C.W.); wehn@eit.uni-kl.de (N.W.); 4 Crop Diseases Research Institute, National Agricultural Research Centre, Islamabad 44000, Pakistan; sofayyaz@yahoo.com; 5 Deep Learning Laboratory, National Center of Artificial Intelligence, Islamabad 44000, Pakistan

**Keywords:** wheat stripe rust disease dataset, semantic segmentation, wheat rust segmentation, computer vision, deep learning

## Abstract

Wheat stripe rust disease (WRD) is extremely detrimental to wheat crop health, and it severely affects the crop yield, increasing the risk of food insecurity. Manual inspection by trained personnel is carried out to inspect the disease spread and extent of damage to wheat fields. However, this is quite inefficient, time-consuming, and laborious, owing to the large area of wheat plantations. Artificial intelligence (AI) and deep learning (DL) offer efficient and accurate solutions to such real-world problems. By analyzing large amounts of data, AI algorithms can identify patterns that are difficult for humans to detect, enabling early disease detection and prevention. However, deep learning models are data-driven, and scarcity of data related to specific crop diseases is one major hindrance in developing models. To overcome this limitation, in this work, we introduce an annotated real-world semantic segmentation dataset named the NUST Wheat Rust Disease (NWRD) dataset. Multileaf images from wheat fields under various illumination conditions with complex backgrounds were collected, preprocessed, and manually annotated to construct a segmentation dataset specific to wheat stripe rust disease. Classification of WRD into different types and categories is a task that has been solved in the literature; however, semantic segmentation of wheat crops to identify the specific areas of plants and leaves affected by the disease remains a challenge. For this reason, in this work, we target semantic segmentation of WRD to estimate the extent of disease spread in wheat fields. Sections of fields where the disease is prevalent need to be segmented to ensure that the sick plants are quarantined and remedial actions are taken. This will consequently limit the use of harmful fungicides only on the targeted disease area instead of the majority of wheat fields, promoting environmentally friendly and sustainable farming solutions. Owing to the complexity of the proposed NWRD segmentation dataset, in our experiments, promising results were obtained using the UNet semantic segmentation model and the proposed adaptive patching with feedback (APF) technique, which produced a precision of 0.506, recall of 0.624, and F1 score of 0.557 for the rust class.

## 1. Introduction

Wheat is a staple crop used across the globe for a variety of food products. A reduction in the supply of wheat can lead to food shortages and malnutrition, especially in developing countries where it is a critical food crop [1]. Wheat stripe rust disease (WRD) is one of the most detrimental diseases of the wheat crop and leads to severe yield losses if untreated. The disease spreads rapidly and affects the entire crop field, leading to severe damage, which, in turn, poses a threat to food security. According to estimates, 88% of the world’s wheat production is susceptible to WRD infection, and 5.47 million tons of wheat are lost to this disease each year [2]. A common approach to detect this disease is the manual inspection of wheat fields by experienced personnel. However, this approach is labor-intensive, slow-paced, and quite inefficient. Alternatively, technology-based solutions are much faster and more accurate. Artificial intelligence (AI)-based methods can be used for farming practices, including early detection of disease, gauging its severity, and predicting the progression of the disease. These AI techniques use deep learning (DL) models to extract meaningful information and features from the underlying data [3]. DL-based algorithms can be used to distinguish between infected and healthy areas in fields [4]. Once a model is trained, the inference parts of the algorithms can be executed on smartphones or drones for real-time monitoring of fields [5]. This detection method can be used to quantify the extent and severity of the disease, enabling early intervention and localized treatment of diseased areas of plants instead of treating the entire crop. However, in order to train these DL models effectively, access to a large, diverse, and well-annotated dataset is crucial. In the literature, there are plant-based datasets available for research, including wheat disease datasets.

Previous research mainly focused on the classification of WRD [5,6,7,8], which is important for early detection. However, semantic segmentation is crucial to estimate the extent of disease spread, which is essential so that the infected plants can be quarantined, remedial measures can be taken, and further spread can be monitored [9]. Unsupervised learning can also be used to extract meaningful information from the data and tackle real-world problems [10]. Driven by these motivations, we propose a semantic segmentation dataset called the NUST Wheat Rust Disease Dataset (NWRD), which allows for the precise localization of disease regions at the pixel level within an image. Furthermore, we perform semantic segmentation using the UNet model, which generates promising results.

Our main contributions are summarized as follows. (1) We present a new multileaf semantic segmentation dataset for WRD. To the best of our knowledge, it is the first real-world multileaf crop field dataset with arbitrarily shaped disease regions, busy backgrounds, occlusions, and varying illumination conditions. Therefore, the images are of high resolution, with a maximum resolution of 6016 × 4000. The only other study related to semantic segmentation was carried out by Yang Li et al. (2022) [11], who presented a segmentation dataset of wheat stripe rust; however, this dataset contained images from a very close-view angle containing only one infected or healthy leaf per image. (2) We evaluated the performance of the abovementioned dataset on a UNet model. Due to the high resolution of the images, we used a downsampling and patching-based approach to reduce the complexity of the dataset. We experimented with grid patching (GP), adaptive patching (AP), and adaptive patching with feedback (APF) techniques. The UNet model with downsampling and APF achieved a 0.506 precision, 0.624 recall, and 0.557 F1 score for the rust class. (3) We demonstrate that the proposed preprocessing and model outperform the state of the art in terms of the F1 score and IoU. (4) We make our dataset, implementation, and pretrained models publicly available at https://github.com/dll-ncai/NUST-Wheat-Rust-Disease-NWRD (accessed on 20 June 2023).

## 2. Related Work

### 2.1. Wheat Disease Datasets

The availability of well-annotated and diverse datasets plays a significant role in the learning of deep learning models [12]. Disease datasets are valuable resources for developing and testing machine learning models for automatic crop disease detection and diagnosis [13]. However, in the literature, open-source wheat disease datasets are scarce. Most of the available wheat disease datasets are classification-based and classify the wheat crops in terms of disease state, type of disease, and disease severity level.

PlantVillage [5], DeepWeeds [14], and PlantDoc [15] datasets are the three most well-known large-scale datasets related to diseases in plants; however, they are not specific to wheat crops. Only a portion of these datasets has images related to wheat diseases. These datasets contain labeled images of crop leaves with different kinds of plant diseases, including some wheat leaf and stem diseases. It is important to mention that all of these are classification datasets, and their main goal is to develop a machine learning model that can classify each image into its respective disease category.

There is a limited number of small-scale wheat datasets available in the literature. Either these datasets are not publicly available or they are not widely adopted. The most recent and well-known wheat disease datasets are presented in Table 1. The table specifies properties related to the datasets, including their name; year of publication and release; the total number of images in the dataset; the type of dataset based on the task that it targets, i.e., classification dataset or segmentation dataset; and the view of images as captured in each image of the dataset. ’Close-view’ images have a single leaf per image captured with no background, a plain/blurred background, or a background with minimal information. However, the ’slight wide-angle view’ captures multiple leaves or an entire plant, along with the background/clutter in the background, making these images much more complex for feature extraction. Another attribute of the dataset is the complexity with respect to the no. of leaves in each image.

The CDTS is the most recent and only research dataset related to the segmentation of wheat rust. This dataset targets the spore and spot segmentation and detection of rust disease. It is also a close-view image dataset, with a focus on spores and spots of rust disease. Every image has a single leaf with diseased and healthy parts, with segmentation masks available for background, diseased, and healthy parts [11]. A rust disease dataset was presented by Shafi et al. (2022) [4]; however, no specific name was assigned to the dataset. It is also a close-view classification dataset of wheat rust, with a single rust leaf with a white background in each dataset image.

The Kaggle research dataset is a freely available classification dataset. The data in this dataset have a close view, with a single leaf per image [6]. The Kaggle Wheat Leaf Dataset is a wheat disease classification dataset with images among one of the following categories: “healthy”, “leaf rust”, “powdery mildew”, or “scab”. The CGIAR crop disease dataset is a WRD dataset with multiple leaves per image. However, this is a classification dataset that classifies diseased wheat leaves between leaf rust and stem rust [7]. The wheat nitrogen deficiency and leaf rust dataset contains controlled and diseased rust leaf images for classification into healthy and diseased images [8].

### 2.2. Wheat Disease Detection Techniques

The problem of wheat head detection and counting from a complex background was addressed by Khaki et al. (2022) [16]. An instance segmentation method, the Hybrid Task Cascade Model, was proposed with Res2Net50 as a backbone network with rich feature learning. The proposed model is a cascaded three-mask RCNN used to construct WheatNet, which learns different features of the plant, thus improving the overall accuracy of detection of wheat spikes in a complex background.

Niu et al. (2014) [17] proposed a segmentation technique for disease detection using the k-means clustering algorithm. The input RGB image of wheat is transformed into the lab color space, and the k-means clustering algorithm is then used to create clusters of high similarity, segmenting the image into foreground (disease) and background. Zhang et al. (2022) [18] proposed that the feature fusion of multiple types of images yields better results for disease detection. Their work also showed that features extracted through deep learning algorithms have much more disease detection accuracy over hand-crafted features. They compared the accuracies of multiple deep learning models, including AlexNet, VGG16, GoogleNet, Xception, ResNet101, etc., and suggested the use of ResNet101 for deep feature extraction due to its deep layers extracting appropriate features for disease identification.

The Mask-RCNN algorithm has also been used to identify the location of wheat rust using semantic segmentation of diseased leaves [19]. Once a large rust leaf is segmented from the background, the pretrained VGG16 and VGG19 classification algorithms are applied to classify the type of wheat disease. However, the background is separated only on the basis of color, and less focused or small rust leaves in the image are not considered. The authors of [20] used ResNet18 to detect disease in high-resolution images, with an accuracy of 77% at the early stage of disease or at the patch level.

Ul Haq et al. (2022) [21] presented an edge AI-based system for classification wheat leaves as healthy or infected. Random forest produced the best accuracy for the binary classification of wheat rust. Using the CGIAR image dataset including leaf rust, stem rust, and healthy wheat images, Sood et al. (2022) [22] applied a pretrained VGG-16 deep-learning-based CNN model to classify the type of rust. They optimized the model to achieve a high training accuracy; however the validation accuracy was 77.14%. Shafi et al. (2022) [4] proposed a framework using machine learning techniques for the precise detection of wheat disease and its infection types. The dataset was gathered from a local field using a mobile camera, where images of individual leaves were taken with a plain white background. Texture features including GLCM and LBP features were extracted from the collected images. Results indicate the combination of the CatBoost algorithm with GLCM texture features produced a classification accuracy of 92.3%. A convolutional neural-network-based model, Yellow-Rust-Xception, was also proposed for the classification of wheat disease into different classes in order to determine the severity level of the disease [23].

Khanfri et al. (2018) [24] discussed the impact of segmentation on the classification accuracy of wheat rust. GrabCut, watershed, and U2-Net segmentation techniques were applied to the dataset, after which the ResNet18 model was used for classification. It was shown that U2-Net segmentation performed the best among the three segmentation models. ResNet18 was then applied to the segmented dataset, and an accuracy of 96.19% was achieved for wheat stripe rust detection.

Image-processing-based techniques have also been used in the literature for assessment of crop damage in wheat fields [25]. Khatra suggested installing cameras in the key areas of the field center, corners, etc., to capture images of the field. Those images are then subjected to image processing techniques to identify growth, as well as the type of disease and infection. Khan et al. (2022) [26] presented an ML-based framework using a fine-tuned RFC model that utilizes decision trees and randomly selects several features from the input features to decide on a class for the input sample in order to identify brown and yellow rust disease in wheat crops. They collected a wheat crop dataset from various fields in Pakistan and applied segmentation and preprocessing techniques to it. The authors of [27] used principal component analysis (PCA) and the Gaussian mixture model to segment wheat lesion images.

Mi et al. (2020) [28] used a wheat rust dataset with six levels of stripe rust infection. They used C-DenseNet, which embeds a convolutional block attention module in the densely connected convolutional network (DenseNet) for this fine-grained image classification problem. The authors of [29] automatically identified the symptom location and disease severity of wheat spikes using dual Mask RCNNs. The authors of [30] used a GAN on the LWCDC2020 wheat disease dataset to predict the missing data in the dataset.

## 3. Materials and Methods

### 3.1. Data Collection Protocol

The wheat crop disease dataset presented in this study is a first-of-its-kind multileaf, open-source segmentation dataset of wheat rust disease collected from crop fields in Islamabad, Pakistan. The data were collected over one season of wheat crop, which was cultivated in November 2021 and harvested in May 2022. Under suitable conditions for rust, the disease starts its onset in February and continues to spread in March and April. The data were progressively obtained during this season in the morning and afternoon.

### 3.2. Data Acquisition—Collection Area

**Figure 1:**
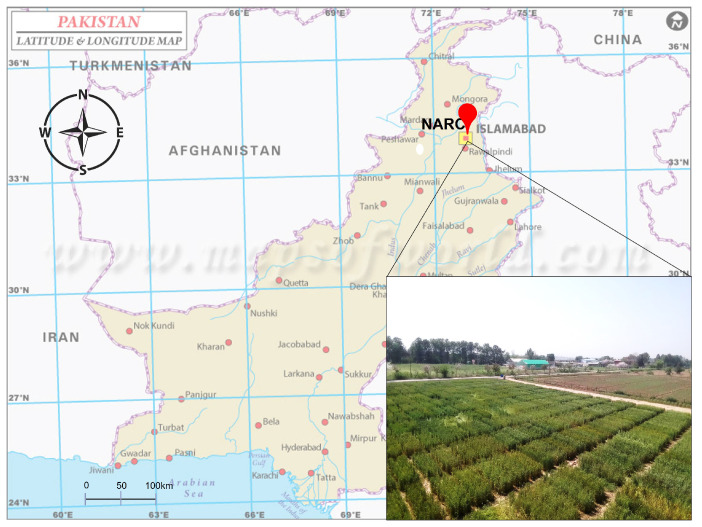
Aerial view of data collection wheat fields at NARC, Islamabad, Pakistan.

The data on wheat rust were collected in collaboration with the National Agriculture Research Centre (NARC), Pakistan. NARC is located six kilometers southeast of the capital, Islamabad. Physical facilities including experimental farm areas, crop fields, and greenhouses are available to undertake the data collection process for research purposes. Figure 1 shows an aerial view of wheat crop fields at NARC where the data were collected.

Images of the diseased and healthy crops were taken under different illumination conditions during the morning and afternoon. The camera-based images collected from the field are of varied resolutions, including a few images of 6016 × 4000 resolution, while the rest have a resolution of 4608 × 3456.

### 3.3. Dataset Properties

The proposed dataset has properties such as high resolution, dense annotation, clear/fuzzy views of disease, arbitrarily shaped disease regions, manual annotation, image complexity, and varied image views. It is a real-world dataset comprising high-quality, high-resolution rust disease images. It clearly portrays the real-world and challenging nature of the diseased crop fields and was captured under natural field and weather conditions. It is a densely annotated dataset with various stages of rust disease (with respect to the spread of disease). Densely annotated images in the dataset comprise multiple diseased rust leaves in a single image.

As it is a real-word dataset, it contains images with clear, fuzzy, and occluded views of WRD. As disease spread on leaves is not symmetric, there are arbitrarily shaped disease regions of rust in each image. This shape variation of disease regions is difficult to annotate due to its arbitrary nature; however, it portrays the true nature of real-world wheat fields.

**Figure 2:**
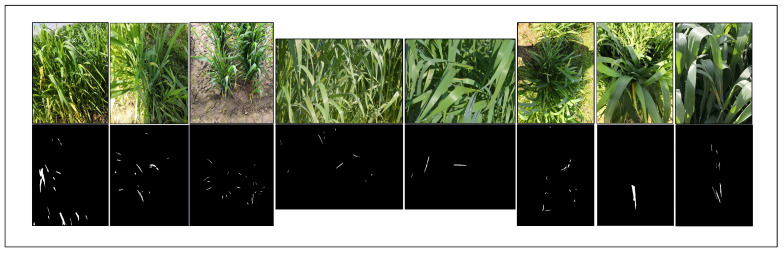
Sample images from the NWRD dataset: annotated images showing rust disease, along with their binary masks.

Ground truths are manually annotated for all instances at a fine-grained level for each image with high precision. Geometric shapes in annotation tools cannot capture the arbitrarily shaped disease regions in the dataset images. Therefore, we categorized the images into two categories with respect to the complexity of the detection of disease, i.e., easy and difficult (close view and slightly wide-angle view, respectively).Furthermore, the dataset presents a varied view of the diseased wheat field. The images capture aerial, as well as ground and front, views of the field, with multiple leaves in a single image. In each image, the majority of the leaves are of the healthy wheat crop class, while a minority of the diseased class creates an imbalance between healthy and diseased leaves. A few annotated sample images from the proposed NWRD segmentation dataset are shown Figure 2, along with their binary masks.

### 3.4. Disease Detection Pipeline for Rust Identification in Wheat Crop

**Figure 3:**
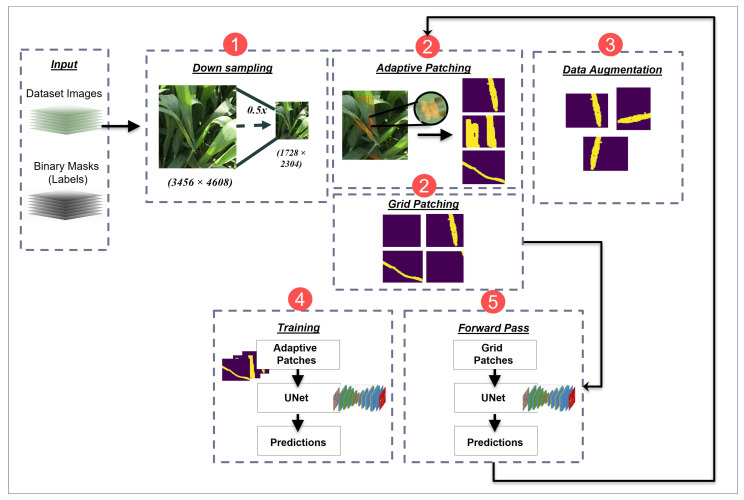
Detailed flow diagram of the training process of the proposed rust disease detection pipeline in wheat crop. Step (1) involves the downsampling of input images to reduce their size. Step (2) is the process of generating patches from input images. It includes both an adaptive patching module and a grid patching module. Step (3) is the data augmentation step at the patch level. Step (4) includes the training process, with adaptive patches as input to the UNet segmentation model, generating predictions. Step (5) is the forward pass that uses grid patches for UNet segmentation and generates prediction masks. These predictions are then sent to the AP module as feedback.

Figure 3 illustrates the disease detection pipeline proposed in this work to extract useful information and features from the NWRD dataset in order to detect WRD in wheat crops.

#### 3.4.1. Downsampling

The images in the dataset are of high resolution relative to the patch size of 128 × 128 pixels that our segmentation model accepts. The area of a patch is very small relative to the total area of the image. This results in the creation of a large number of patches per image. A possible solution to reduce the number of patches is to increase the patch size; however, increasing the patch size also increases the number of computations. Another solution to this problem is to downsample the input images beforehand to a smaller size and use them for training. Downsampling is the reduction in the spatial resolution of an image. This process reduces the overall number of patches, reducing the training time.

The process of downsampling results in a slight decrease in the F1 score, but this is a tradeoff with the benefit of faster training in the case of a large dataset. Downsampling can be performed using multiple methods; each method preserves information about lost pixels in a different way. For our experiments, we used Lanczos filtering, which uses a modified sinc filter to preserve the spatial information as much as possible. Lanczos filtering is a technique that is used to resize images while maintaining their visual quality. This is achieved by calculating new pixel values of the resized image based on a weighted average of neighboring pixels of the original image. The weights are calculated using a mathematical function known as the Lanczos kernel. The Lanczos kernel is defined as:

(1) $$L\left(x\right)=\left\{\begin{array}{cc} \sin c\left(\pi x\right)\sin c\left(\frac{\pi x}{a}\right), & \mathrm{if}\;-a\leq x\leq a\\ 0, & \mathrm{otherwise} \end{array}\right.$$

where sinc(πx) is the sinc function defined as sin(πx)/(πx), and *a* is a parameter known as the filter radius, which governs the size of the kernel used in resizing an image. It determines the extent to which neighboring pixels affect the value of each pixel in the resized image. This method effectively reduces aliasing artifacts and preserves the details and sharpness of the original image [31].

#### 3.4.2. Image Patching

The real-world images that are captured containing diseased and healthy leaves of wheat are high-resolution images. The processing of these images is intensive and time-consuming. As a first step of preprocessing, we break down these images into multiple patches to feed to the model. We use a patch size of 128 × 128 pixels for both the images and binary masks. This means that we can directly train our segmentation model on these patches.

We explored three different patching techniques: grid patching (GP), adaptive patching (AP), and adaptive patching with feedback (APF). The GP technique is used to divide a larger image into a grid of smaller patches. These patches do not overlap with each other. This technique is the most commonly used patching technique when working with high-resolution images in deep learning. The patches can then be easily preprocessed individually and used to train the convolutional models. However, the grid patching technique comes with a drawback: it generates a large number of patches. When a dataset has an imbalance between classes, like the NWRD dataset with imbalanced rust and non-rust classes, grid patching generates a lot of unnecessary patches that contain non-rust parts but do not contain rust parts. In this case, training segmentation models with GP comparatively takes more time training on the non-rust class than on the rust class and does not contribute much to the overall performance of the model. This unnecessary training on the non-rust part also results in an increase in the overall time taken by the training process.

The AP technique is used to select the most useful patches for training from an image while ensuring that patches with the maximum information are extracted. This technique is used to reduce the number of patches that are input to the model, consequently reducing the overall training time of the model. Unlike grid patching, this technique of adaptive patching dynamically selects patches on runtime during training as required by the model.

**Figure 4:**
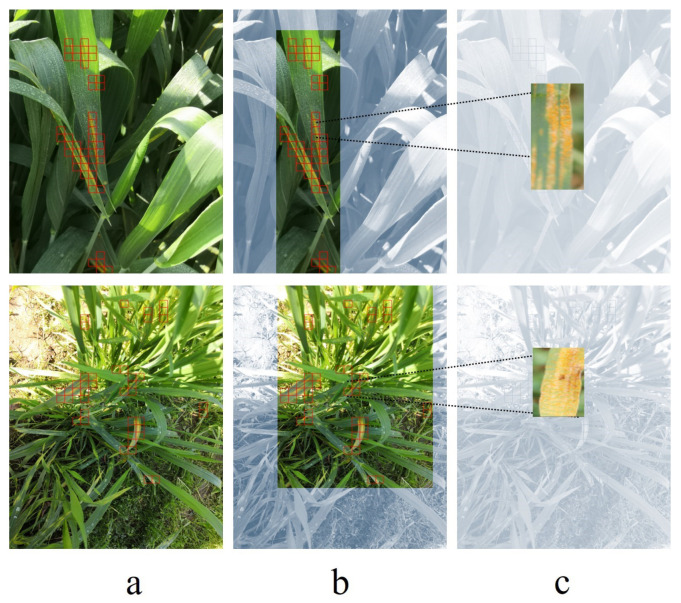
The process of obtaining patches from an image. (**a**) Sample images from the dataset, with red boxes indicating the presence of rust. (**b**) Section of the image with rust-effected leaves (**c**) Final patches from the sample image, which are fed to the model for prediction.

Adaptive patching selects only those patches from the images for training that contain specs of rust in them. The rest of the patches from the image are discarded. In Figure 4, the red boxes represent patches from the image with rust on the leaves. The threshold value that we set for a patch to be selected for training is 1%, which means that a patch with at least 1% of rust pixels with respect to the overall pixels in an image will be selected in the process of adaptive patching. In this technique, the model is selectively trained on only rust patches, as the rest of the image patches are discarded.

The images from the NWRD dataset show that the ratio of rust and non-rust area within an image is not balanced. This introduces the problem of unbalanced data for rust and non-rust patches. If only adaptive patching is used where rust-based patches are input to reduce the computations, the model becomes biased towards rust and generates false positives on test images. To minimize this problem, we propose a new technique of adaptive patching with feedback in which rust and noise-based patches from the input images are selected for feature learning.

The adaptive patching with feedback technique has two main steps. (1) It starts with a simple adaptive patching technique as discussed in the section above. It selects a few patches of the image for training that contain some traces of rust and discards other patches. This reduces the total number of patches by almost 10 times, consequently reducing the training time. It is noteworthy that only patches of a single class are obtained by this method, i.e., the disease class. Each rust patch obtained by this selective patching technique contains 128 × 128 pixels.

(2) As a second step, a feedback loop is used, i.e., model predictions are merged with the target binary masks of input images in order to include noisy patches for training. The new mask then contains both areas that are annotated as rust in the original masks and areas that the model segmented as rust. This means that the adaptive patching algorithm can now pick up the pixels in the image that were part of the rust traces and the pixels that were classified as false positives by the model. The algorithm then includes patches according to the new mask for the upcoming training epochs. This trains the model in areas where rust is present, as well as in areas where the models struggled in the previous epochs. The training continues, with step 2 executed after constant intervals.

#### 3.4.3. Data Augmentation

**Figure 5:**
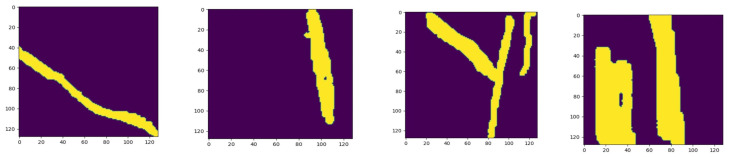
Patches generated from the original images of the NUST Wheat Rust Disease Dataset (NWRD). The yellow area in a patch indicates the presence of rust disease, while the purple area shows the non-rust part of a patch.

In the deep learning paradigm, the more data, the better; therefore, data augmentation is useful to increase the size of a dataset by generating new variations of existing data samples. This will help with improving the accuracy and generalization capabilities of the detection model. Cheng et al. (2023) [32] proposed a new augmentation model to address the dataset scarcity problem for wheat disease detection. In our work, we applied patch-level augmentation, including horizontal and vertical flips of the patches passed down to the data augmentation module through the patching module, as illustrated in Figure 5.

Table 2 shows the total number of patches generated using the grid patching and adaptive patching techniques after the data augmentation step. As we input 22 and 100 images of the NWRD dataset, for training specifically, there is a huge difference between the number of generated patches. Adaptive patching definitely reduces the number by ensuring that patches with maximum useful information are retained for training.

#### 3.4.4. Segmentation

A UNet model with 4 encoders and 4 decoder blocks was trained with the preprocessed dataset. The model input size was set to 128 × 128 pixels. Images were split into patches with dimensions of 128 × 128 to be fed into the model for training. The model was trained with patch strides of different sizes. From the experimentation results, it was observed that the model did not perform best with a patch stride of 128. However, a patch stride of 32 appeared to have a good balance between the overall number of generated patches and the time taken for the training process to complete.

#### 3.4.5. Forward Pass

During the training process, a forward pass is used to update the weights of the UNet segmentation model, and another forward pass is used for the purpose of feedback. The first forward pass uses the patches generated through adaptive patching; those patches are then passed through the data augmentation module to apply augmentation techniques to the patches. Once augmentation is done, in this forward pass, focal loss is used to update weights during backpropagation. The second forward pass uses grid patches obtained from the training dataset and generates prediction masks for the training data. The prediction masks are then merged with the target binary masks to form a new mask, which is then used by the adaptive patching algorithm to generate patches for the upcoming epochs. However, the second forward pass occurs after a fixed number of training epochs. In our experimentation, we observed that 5 epochs between the adaptive patching process yield better results in providing a good balance between time complexity and accuracy of the UNet segmentation model on the NWRD data.

## 4. Training and Results

### 4.1. Performance Evaluation Metrics

The performance of the segmentation model for wheat rust detection was evaluated using well-known evaluation metrics. The F1 score is the harmonic mean of precision and recall. It is a single metric that summarizes both precision and recall. The NWRD dataset presents images in which the majority is non-rust or healthy and a small portion comprises rust leaves. This means that there is a class imbalance problem prevalent in our data, while dealing with class-imbalanced data, choosing evaluation metrics sufficiently robust to class distribution is essential. F1 score is a helpful evaluation metric for imbalanced data because it provides a balanced measure of precision and recall. The F1 score is calculated using the following formula:

(2) $$F1\text{-}\mathrm{score}=2\cdot \frac{\mathrm{Precision}\cdot \mathrm{Recall}}{\mathrm{Precision}+\mathrm{Recall}}$$

Precision refers to the proportion of correctly identified rust disease cases (i.e., true positives) among all the predicted rust disease cases. In other words, it measures how accurate the model is when it predicts that a wheat crop has rust disease. A high precision value means that the model is making fewer false-positive errors, i.e., predicting that a wheat crop has rust disease when it actually does not.

(3) $$\mathrm{Precision}=\frac{\mathrm{True}\;\mathrm{Positives}}{\mathrm{True}\;\mathrm{Positives}+\mathrm{False}\;\mathrm{Positives}}$$

Recall, on the other hand, refers to the proportion of correctly identified rust disease cases among all the actual rust disease cases. In other words, it measures how well the model is able to identify all the wheat crops that actually have rust disease. A high recall value means that the model is making fewer false-negative errors, i.e., correctly identifying most of the wheat crops that have rust disease.

(4) $$\mathrm{Recall}=\frac{\mathrm{True}\;\mathrm{Positives}}{\mathrm{True}\;\mathrm{Positives}+\mathrm{False}\;\mathrm{Negatives}}$$

### 4.2. Model Training

The overall training process of UNet is divided into two stages: model training and a forward pass to generate predictions of the GPs for feedback. The preprocessing and the UNet model are implemented in Python and Pytorch, respectively. In our experiments, we explored the aforementioned performance parameters on 22 and 100 images of the dataset. The numbers of patches generated for the GP and AP are presented in Table 2. The images are randomly selected, with 80% assigned to training, 10% to validation, and 10% to testing.

**Figure 6:**
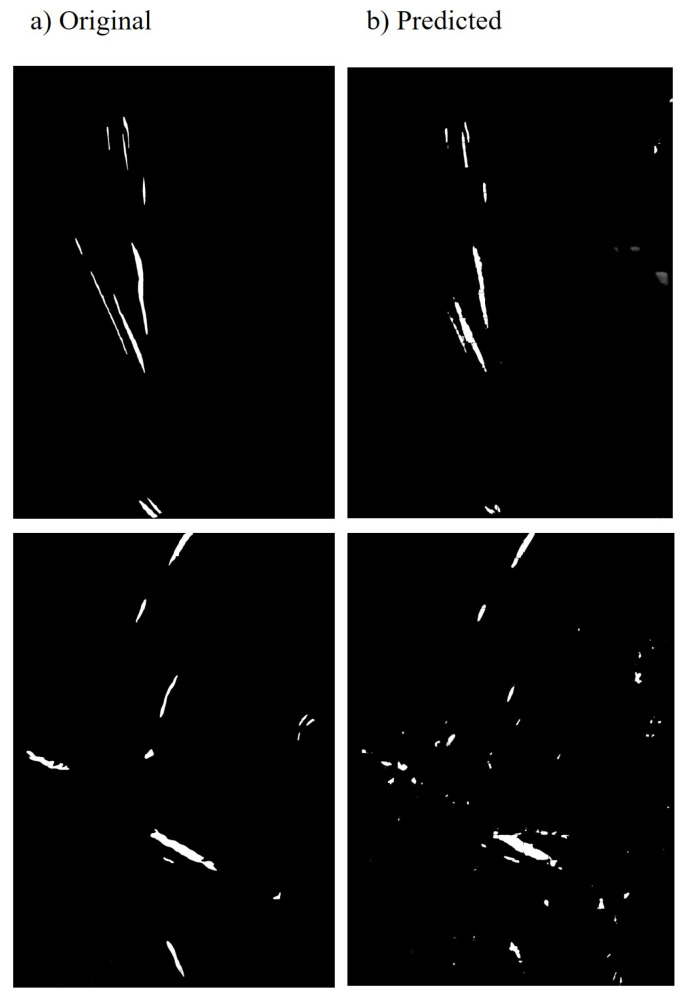
Qualitative analysis: the visual results of rust detection. (**a**) The original binary mask of diseased leaves. (**b**) Results predicted by the rust detection model.

For UNet model training, we used the *RMSprop* optimizer [33], with the initial learning rate set to $1\times 10^{-6}$ and scheduled to decrease exponentially using the *ExponentialLR* scheduler. Training was carried out for 500 epochs, utilizing a batch size of 64 and a stride of 32 and 128. Furthermore, we experimented with multiple loss functions, such as *focal loss* and *dice loss*. In our case, focal loss yielded the best results. The experiments were conducted on two NVIDIA Tesla V100 GPUs, each with 32 GB memory. The best qualitative results for disease detection in wheat crops using the NWRD dataset are shown in Figure 6.

### 4.3. Results

Table 3 presents the experimental results for different patching techniques and input strides on the test dataset for 22 and 100 images. Initially, we experimented with a smaller dataset (22 images) to estimate the hyperparameters for compression of the input images and the UNet training and later implemented the complete dataset. For the smaller dataset, GP with a stride of 32 generated the best results by achieving 0.683 precision, 0.685 recall, and 0.684 F1 score for the rust class. However, from the training perspective, it took approximately 11 days to train the model. In contrast, APF with an input stride of 32 achieved an F1 score of 0.65 after approximately 2 days of training. The training time column in Table 3 refers to the total time taken (in minutes) by the semantic segmentation pipeline using different patching techniques, numbers of images, and input strides to yield the results. Here, we report the model training time instead of GPU hours, since preprocessing is performed on the CPU and the model is trained on the GPU. For the complete dataset, APF with a stride 32 achieved 0.506 precision, 0.624 recall, and 0.557 F1 score for the rust class. For a fair comparison, we also trained a model for GP with an input stride of 32; however, the training did not complete due to the high memory utilization of the GPU.

## 5. Discussion

Datasets are instrumental in addressing complex research questions [34], and they play a vital role in advancing research across various disciplines [35]. The availability of high-quality datasets is crucial for pattern and trend analysis, driving research advancements [36]. In this work, we present a challenging open-source segmentation dataset of WRD. This is a multileaf complex dataset of WRD with slightly wide-angle images of wheat crops. It is challenging due to the high complexity of the images. The dataset has arbitrarily shaped disease regions, is densely annotated, and includes complex foregrounds and backgrounds. Semantic segmentation helps in fine-grained understanding of the structure and layout of images and finds its applications in various computer vision tasks [37]. Semantic segmentation is also utilized for disease identification and detection in plants and fruits, which is a tedious and complex task [38]. Training of segmentation models requires a vast amount of data to learn patterns and trends. The open-source dataset presented in this work will bridge the gap between the wheat disease detection problem and deep learning solutions by solving the data scarcity problem in this domain.

The only semantic segmentation research work related to wheat disease segmentation was carried out by Li et al. (2022) [11]. In their research, the authors concluded that Octave-UNet outperformed UNet and PSPNet on the CDTS dataset in terms of IoU over a small region and improved accuracy of spore and leaf segmentation. To compare our proposed technique with state-of-the-art work, we trained and tested UNet and Octave-UNet segmentation models on the proposed NWRD dataset, along with the APF patching technique. For a fair comparison, we used the same preprocessing techniques proposed by Li et al. (2022) [11] (images containing only background were removed from the train and test datasets). Additionally, similar hyperparameters for training were used, such as a stride of 128, to generate the evaluation metrics of precision, recall, F1 score, and IoU. Table 4 shows a comparison of the results of our proposed techniques with those of the state of the art in the field of WRD detection.

The results show that the APF technique with the UNet segmentation model achieves an F1 score of 0.564 and an IoU of 0.438, while Octave-UNet with the APF technique yields an F1 score of 0.529 and an IoU of 0.316. The results clearly demonstrate that in the case of the NWRD dataset, which is a complex real-world WRD dataset, the APF patching technique with the UNet segmentation model outperforms Octave-UNet in terms of F1 and IoU scores.

A comparison of our approach of APF and the UNet segmentation model with the Octave-UNet model indicates that for the real-world NWRD dataset, our technique performs better than the state-of-the-art Octave-UNet model. In their wheat segmentation work, the authors used an image filtering technique that discards all image patches with a pure background, i.e., no rust spores or spots. We argue that removing the background images from the dataset hinders the ability of the model to learn the actual trend of the onset of the disease. Consequently, the model will generate false positives when tested on real-world WRD field data. The early onset of WRD affects a minority of leaves while the majority of the leaves in the field are healthy. The model also needs to train on healthy images to generalize and distinguish between rust and non-rust parts more accurately.

In conclusion, the UNet segmentation model, along with the APF technique, considerably reduces the time taken for training and produces comparable results. Hence, there lies a tradeoff between processing time and precision. Since the rust spots, as compared to the healthy part of the leaf in most of the images, are very small, the UNet model is not performing at its best. Our results demonstrate that a semantic segmentation approach can be used to estimate the infected area in a wheat field. The initial results of the segmentation are promising, with room for further improvement. This is the main motivation behind making the NWRD dataset public so that the research community can explore models and techniques to solve the problem of controlling WRD disease in wheat crops.

The investigations carried out in this study will lead to new opportunities for crop monitoring—specifically segmentation of WRD in wheat crops. Since WRD is infectious, the segmentation algorithm will help estimate the infected plants in their initial stages. Furthermore, the open-source pretrained models can be mapped to other diseases in crops, such as ascochyta blight in cotton and bacterial leaf streak disease in corn, using the transfer learning approach. One of the limitations of our work is the class imbalance problem prevalent in the NWRD dataset. As a complex dataset focusing on the early onset of rust disease, the captured images contain a major portion of healthy leaves and a minor percentage of disease, resulting in the requirement of a multistage preprocessing and feature extraction process. In the future, we plan to improve the preprocessing module and the detection pipeline, which will consequently improve the segmentation results.

## 6. Conclusions

In this work, we present an open-source semantic segmentation dataset of WRD named the NWRD. The dataset contains high-resolution (max. 6016 × 4000) multileaf images from wheat fields under various illumination conditions with complex backgrounds. Previous works in this domain have focused on single-leaf, low-resolution images. Since the input image size is quite large, we explored different compression techniques, such as down-sampling and patch-based segmentation, to reduce the memory footprint. We also implemented the UNet model with different patching strategies for semantic segmentation of the NWRD. We achieved 0.506 precision and a 0.557 F1 score for the rust class. We also compared the presented approach with state-of-the-art segmentation techniques specifically in the area of wheat disease segmentation. Our proposed preprocessing technique of adaptive patching with feedback (APF) along with the UNet model showed an improved F1 score and IoU as compared to Octave-UNet on the NWRD dataset. The achieved segmentation results are promising, with room for further improvement. Therefore, we made all explorations, models, and implementations open-source so that our approach can be easily adopted or further improved by the research community. In future work, we intend to improve the disease detection pipeline by applying fine-grained feature extraction techniques that can consequently improve the segmentation results.

## Figures and Tables

**Table 1:** Comparison of available wheat disease datasets.

No.	Dataset	Year	# of Images	Type	Image View	Image Complexity
1.	Wheat Yellow Rust Disease Infection type Classification [4]	2021	268	Classification	Close view	Single leaf
2.	Kaggle Wheat Leaf Dataset [6]	2021	407	Classification	Close view	Single leaf
3.	CGIAR Computer Vision for Crop Disease Dataset [7]	2020	1486	Classification	Close view	Multiple leaves
4.	Wheat Nitrogen Deficiency and Leaf Rust Image Dataset [8]	2020	859	Classification	Close view	Single leaf
5.	Crop Disease Treatment Dataset (CDTS) [11]	2022	2353	Segmentation	Close view	Single leaf
6.	NUST Wheat Rust Disease Dataset (NWRD) (This Work)	2023	100	Segmentation	Slightly wide-angle view	Multiple leaves

**Table 2:** Breakdown of the patches generated using grid and adaptive patching techniques for input images of NWRD during the process of training, testing, and validation.

Images	Process	No. of Patches Generated in GP (Stride 32)	No. of Patches Generated in AP
	Training	72,592	average of 12,000
22	Validation	468	468
	Test	703	703
	Training	327,929	average of 80,000
100	Validation	2237	2237
	Test	2737	2737

**Table 3:** The semantic segmentation results of the 22 and 100 images of the WRD with different patching techniques and input strides using the NWRD dataset. The experiments were conducted on two NVIDIA Tesla V100 GPUs, each with 32 GB memory.

Patching Type	# of Images	Input Stride	Precision	Recall	F1 Score	Training Time (mins.)
GP	22	128	0.694	0.557	0.618	1676
GP	22	32	0.683	0.685	0.684	15,820
APF	22	128	0.685	0.555	0.613	743
APF	22	32	0.578	0.743	0.650	2661
GP	100	128	0.510	0.544	0.514	7474
APF	100	32	0.506	0.624	0.557	4791

**Table 4:** Comparison of our proposed work with the state-of-the-art semantic segmentation techniques.

Technique	Model	Input Stride	Precision	Recall	F1 Score	IoU	Training Time (mins.)
APF	Octave-UNet	128	0.580	0.497	0.529	0.316	923
APF	UNet	128	0.593	0.552	0.564	0.438	678

## Data Availability

The NWRD dataset proposed in this research work, along with the pretrained models, can be accessed through this link: https://github.com/dll-ncai/NUST-Wheat-Rust-Disease-NWRD (accessed on 20 June 2023).

## Abbreviations

The following abbreviations are used in this manuscript:

WRD	Wheat stripe rust disease
NWRD	NUST wheat rust disease dataset
AI	Artificial intelligence
NARC	National Agriculture Research Centre
SEECS	School of Electrical Engineering and Computer Science
SMME	School of Mechanical and Manufacturing Engineering
NUST	National University of Sciences & Technology
NCAI	National Center of Artificial Intelligence
GP	Grid patching
AP	Adaptive patching
APF	Adaptive patching with feedback
